# An enrichment method to increase cell-free fetal DNA fraction and significantly reduce false negatives and test failures for non-invasive prenatal screening: a feasibility study

**DOI:** 10.1186/s12967-019-1871-x

**Published:** 2019-04-11

**Authors:** Ping Hu, Dong Liang, Yangyi Chen, Ying Lin, Fengchang Qiao, Hang Li, Ting Wang, Chunfang Peng, Donghong Luo, Hailiang Liu, Zhengfeng Xu

**Affiliations:** 1 State Key Laboratory of Reproductive Medicine, Department of Prenatal Diagnosis, Obstetrics and Gynecology Hospital Affiliated to Nanjing Medical University, Nanjing Maternity and Child Health Care Hospital, Nanjing, Jiangsu China; 2 CapitalBio Technology Inc., Beijing, 101111 China; 3 The Affiliated Suzhou Hospital of Nanjing Medical University, Suzhou, 215000 Jiangsu China; 4 CapitalBio Genomics Co., Ltd., Dongguan, 523808 China; 5 CapitalBio MedLab, Beijing, 102206 China

**Keywords:** NIPS, cfDNA screening, cffDNA enrichment, cffDNA fraction, False negative, Test failure

## Abstract

**Background:**

Noninvasive prenatal screening (NIPS) based on cell-free fetal DNA (cffDNA) has rapidly been applied into clinic. However, the reliability of this method largely depends on the concentration of cffDNA in the maternal plasma. The chance of test failure results or false negative results would increase when cffDNA fraction is low. In this study, we set out to develop a method to enrich the cffDNA for NIPS based on the size difference between cell-free DNA (cfDNA) of fetal origin and maternal origin, and to evaluate whether the new NIPS method can improve the test quality.

**Methods:**

We utilized 10,000 previous NIPS data to optimize a size-selection strategy for enrichment. Then, we retrospectively performed our new NIPS method with cffDNA enrichment on the 1415 NIPS samples, including 1404 routine cases and 11 false negative cases, and compared the results to the original NIPS results.

**Results:**

The 10,000 NIPS data revealed the fetal fraction in short cfDNA fragments (< 160 bp) is significantly higher. By using our new NIPS strategy on the 1404 routine cases, the fetal fraction increased from 11.3 ± 4.2 to 22.6 ± 6.6%, and the new method performed a significant decrease of test-failure rate (0.1% vs 0.7%, P < 0.01). Moreover, in 45.5% (5/11) of the false negative cases, fetal trisomies were successfully detected by our new NIPS method.

**Conclusions:**

We developed an effective method to enrich cffDNA for NIPS, which shows an increased success rate and a reduced chance of false negative comparing to the ordinary NIPS method.

**Electronic supplementary material:**

The online version of this article (10.1186/s12967-019-1871-x) contains supplementary material, which is available to authorized users.

## Background

The discovery of cell-free fetal DNA (cffDNA) in maternal plasma by Lo et al. in 1997 has inspired various non-invasive prenatal screening (NIPS) applications [1], which avoids the ~ 1:100 chance for miscarriage introduced by invasive sampling. At present, NIPS for common fetal aneuploidies, based on analysis of cffDNA in maternal plasma, has been gradually applied as a first-tier aneuploidy screening strategy in clinical practice [2, 3]. Previous large-scale clinical studies have revealed high accuracy of NIPS in screening on trisomy 21, 18 and 13, with sensitivity and specificity higher than 95% [4–6].

Importantly, the reliability of NIPS largely depends on the assumption that there is sufficient fetal DNA in the samples tested [7]. Fetal fraction, the percentage of cell-free DNA (cfDNA) that is from fetal origin in maternal peripheral blood, is generally at the range of 3–30%, with an average of about 13% [4]. The cffDNA levels are determined by multiple factors, including gestational age, maternal weight and extraction method [8, 9]. In addition, fetal fraction could further decrease during sample transportation or laboratory work-up in NIPS. Previous researches have shown that the extent of chromosomal abnormalities presented in plasma of women with aneuploidy pregnancies is linearly correlated with the cffDNA fraction, thus the test accuracy of NIPS largely relies on the fetal fraction [10]. Most current NIPS protocols utilize 4% as the lower fetal fraction cutoff value to ensure a reliable result. However, for pregnancies at early GA stages or obese women who require NIPS, low fetal fraction is the major issue to overcome [8]. In addition, NIPS misses about 1% chromosomal aneuploidy cases, and the most common factor associated with these false negative results is the low fetal fraction [11, 12]. As for the aforementioned reasons, it is critical to elevate fetal fraction for achieving convincing NIPS results.

cfDNA is DNA fragments generated from apoptotic cells, which is released into circulation after rapid DNA degradation. The size distribution of these DNA fragments has peaks corresponding to nucleosomes (~ 143 bp) and chromatosomes (nucleosome + linker histone; ~ 166 bp) [13, 14]. In pregnant women, cffDNA in maternal peripheral blood mainly originates from placental trophoblasts, crossing through the placental barrier [1]. In 2010, Lo et al. found that cffDNA exhibits a different length distribution comparing to the cfDNA from maternal cells, with a reduced proportion of molecules of 166 bp and an increased proportion of molecules of shorter than 150 bp in maternal plasma, possibly caused by differential nucleosomal packaging during apoptosis, or by differences in the force of nucleosome binding [15]. Based on these findings, it is theoretically possible to enrich cffDNA fragments from total cfDNA in maternal peripheral blood by size selection.

In the present study, we set out to develop an experimental method for cffDNA enrichment, which increased the mean cffDNA fraction by 1.5–4 times, while the complexity of cfDNA obtained is fully adequate for NIPS. Moreover, we retrospectively tested 1415 clinical samples including 1404 routine clinical NIPS samples and 11 false negative NIPS samples using this new method. Our results demonstrated the cffDNA enrichment strategy can improve the overall performance of NIPS by reducing false negative results as well as the test failure rate.

## Methods

### DNA extraction and libraries construction

Five to 10 mL of peripheral venous blood was collected from each participating pregnant woman in EDTA-containing tubes or Streck blood collection tubes. The blood samples were first centrifuged at 1600×*g* for 10 min at 4 °C to separate the plasma from peripheral blood cells. The plasma portion was carefully transferred to a polypropylene tube and subjected to centrifugation at 16,000×*g* for 10 min at 4 °C to pellet the remaining cells. Cell-free DNA from 600 μL of maternal plasma was extracted using the QIAamp DSP DNA Blood Mini Kit (Qiagen) following the blood and body fluid protocol. End-repairing, adaptor ligation and PCR amplification were performed using Ion Plus Fragment Library Kit (Life Technologies).

### Cell-free fetal DNA enrichment

DNA enrichment was performed after end-repairing and before adaptor ligation during NIPS library construction. Magnetic beads with an average particle size of 1 μm were used for the purpose of size-selecting the end-repaired DNA fragments with size smaller than 160 bp. To achieve the highest efficiency, we optimized this step by testing a series of different bead concentrations. Magnetic beads were added to the end-repaired DNA fragments, followed by vibrating the tubes for at least 3 s. The tubes were then suspended for 5 min and transferred to a magnetic rack. The supernatant containing size-selected DNA fragments was then transferred to another tube for adaptor ligation.

### Sequencing

DNA library concentration was determined by Qubit and RT-PCR. For DNA sequencing, 15–20 libraries were pooled and sequenced using JingXin BioelectronSeq 4000 System (CFDA registration permit NO. 20153400309) semiconductor sequencer with single-end sequencing mode in 400 flows producing raw sequencing reads with size of up to 200 bp and counts of at least 3.5 million.

### Data analysis

Reads trimmed from the 3′ end by sequencing quality value of > 15 and filtering by reads with length of shorter than 50 bp were aligned to the human genomic reference sequences (hg19) using the BWA [16]. Reads that were unmapped or had multiple primary alignment records were filtered by FLAG field in the alignment file, using an in-house Perl script. Duplicate reads were identified by Picard (http://picard.sourceforge.net/). The remaining reads were considered unique reads for further analysis. To eliminate the effect of GC bias, we calculated the number of unique reads for each 20 kb-bin, then applying an integrated method for GC correction using a three-step process: Locally weighted scatterplot smoothing (LOESS) regression [17], intrarun normalization [18], and linear model regression [19]. LOESS regression was performed in R software with default parameters. We derived a z score for each of the chromosomes in a test sample by subtracting the mean chromosome ratio in a reference set of euploid control pregnancies from the chromosome ratio in a test case and dividing by the SD of the chromosome ratio in the reference set according to the following equation: a cutoff value of z score > 3 was used to determine whether the ratio of the chromosome was increased and hence fetal trisomy was present.

### Estimation of cffDNA fraction

Two types of methods were used to calculate the fetal DNA fraction in maternal plasma. For one method, the cffDNA fraction for pregnancy with a male fetus can be easily estimated using reads proportion on the Y chromosome. For the other method, the cffDNA fraction can be estimated using length distribution of cffDNA. Fetal DNA is generally shorter than maternal DNA, Plasma samples with a higher fetal DNA fraction would have a higher proportion of short plasma DNA fragments (~ 130–140 bp; region A) and a lower proportion of long plasma DNA fragments (~ 155–175 bp; region B). LOESS regression was applied to fit the fetal fraction against reads ratio in features A and B. We obtained the LOESS fit-predicted fetal fraction P_A_ for feature A and P_B_ for feature B. Because both P_A_ and P_B_ predict the fetal DNA fraction, P_A_ and P_B_ should also closely correlate. Therefore, we used reference samples to compare P_A_ and P_B_ and thus, identify instances of poor correlation. If P_diff_ = (P_A_ − P_B_) × 2/(P_A_ + P_B_) is larger than 0.40 (larger than 99% normal samples), P_A_ and P_B_ are inconsistent, and the fetal fraction is considered unpredictable. Otherwise, we calculated the final predicted fetal fraction using P = (P_A_ + P_B_)/2.

### DNA complexity calculation

In order to evaluate the DNA loss during the enrichment procedure, the amounts of sequencing libraries with or without enrichment were calculated using qPCR and KAPA Library Quantification Kit. The qPCR with primers of 5′-CCTCTCTATGGGCAGTCGGTG-3′ and 5′-CCTGCGTGTCTCCGACTCAG-3′ was performed using SYBR Green Realtime PCR Master Mix (TOYOBO). Besides the amounts, DNA complexity was a major factor for PCR amplification and sequencing. Lower DNA complexity would result with higher percentage of duplicated reads when sequencing, leading to the test failures of NIPS. Thus, we applied a method of captured sequencing covering 300 SNPs for libraries constructed with and without beads enrichment. Capture probes (data not provided) were designed and synthetized by Agilent. DNA hybridization and sequencing were performed according to the manufacturer’s instructions. After reads mapping and SNP calling, DNA complexities were calculated using the following equation:

$$ DNA \;complexity = \frac{{   {\text{unique reads covered SNPs in design }}}}{\text{SNP number in panel}}. $$

### NIPS sample cohort

10,000 NIPS data, 1404 regular NIPS cases and 11 false negative cases were recruited in this study. All participants signed informed written consent before blood collection. This study was approved by the institutional review board of the Affiliated Obstetrics and Gynecology Hospital of Nanjing Medical University.

### Confirmation of original NIPS results

Pregnancies with positive NIPS results were recommended for confirmatory invasive prenatal diagnosis using amniocentesis following karyotyping and/or chromosomal microarray analysis (CMA). Pregnancies with negative NIPS results were interviewed at 3 months after delivery to record the information, including the ultrasound examination, pregnancy outcomes, newborn physical examination results, and neonatal/fetal cytogenetic analysis.

### Statistical analysis

Statistical analysis between the different groups was performed using a Chi square (X^2^) test or Fisher’s exact test, and P-values of ≤ 0.01 were considered statistically significant.

## Results

### Optimizing size-selection strategy for cffDNA enrichment

Based on the hypothesis that the fetal fraction are correlated with size distribution of the cfDNA fragments, we set out to develop a method that can improve cffDNA fraction by selectively enriching short cfDNA fragments.

**Fig. 1:**
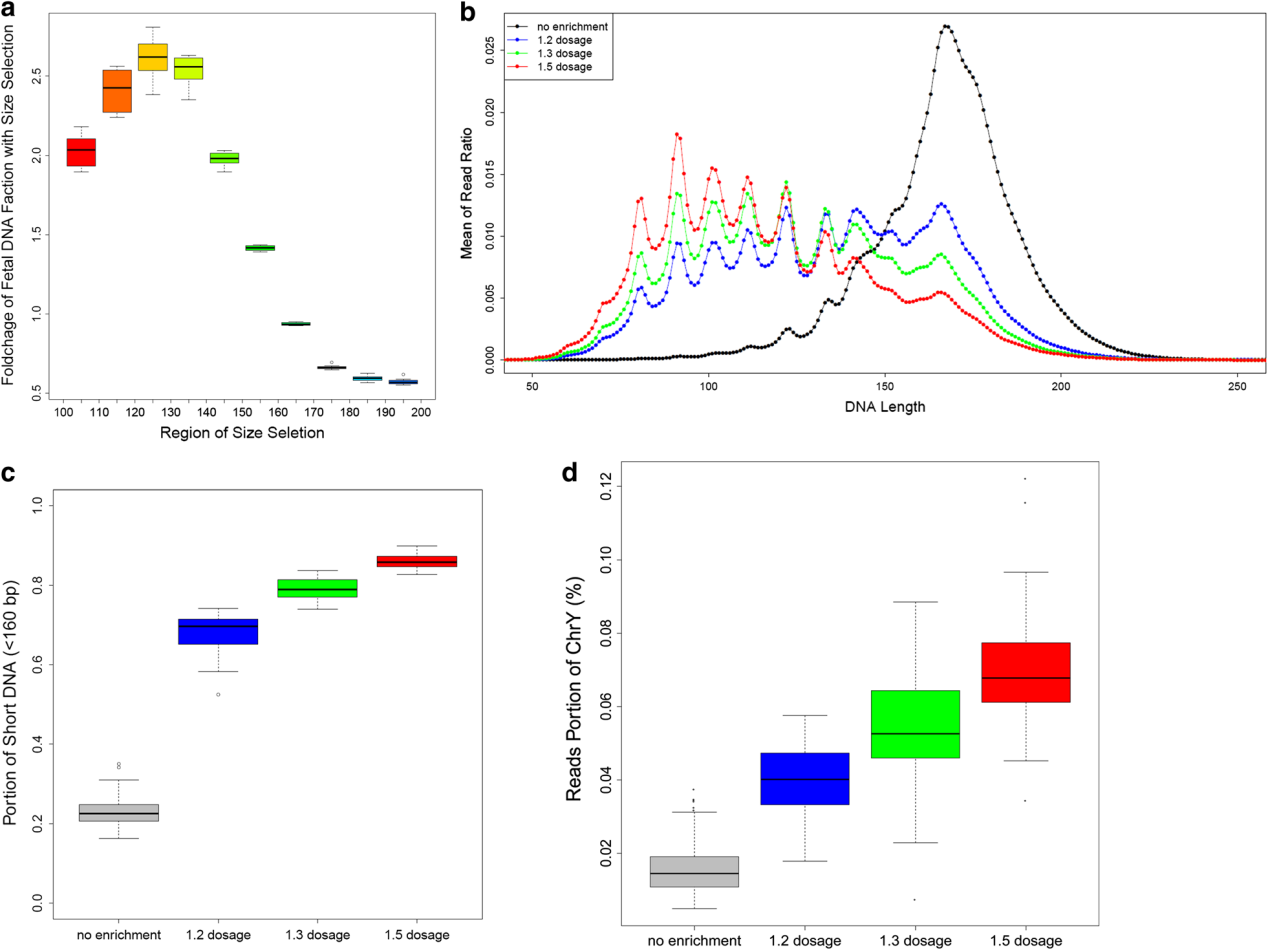
The effect of cffDNA enrichment. **a** Foldchange of cffDNA fraction in 10 different bins, including [100, 110), [110, 120), [120, 130), [130, 140), [140, 150), [150, 160), [160, 170), [170, 180), [180, 190), [190, 200), calculated using reads portion of the Y chromosome. **b** Alteration of read length distribution after size-selection using our customized beads with different dosages. **c** The portion of short reads (< 160) increased after size-selection using our customized beads with different dosages. **d** The Z-score of ChrY increased after size-selection using our customized beads with different dosages

In order to investigate the correlation between cfDNA fragment size and cffDNA fraction, we collected a total of 10,000 existing NIPS data from pregnancies with male fetus. We grouped data from every 100 samples into one mixed data set, generating a total of 100 mixed data sets. The data set was produced by single-end sequencing mode with read length of 200 bp and read counts of 3.5 million using JingXin BioelectronSeq 4000 System. For each mixed data set, reads were divided into 10 different bins according to their sequence length, including [100, 110), [110, 120), [120, 130), [130, 140), [140, 150), [150, 160), [160, 170), [170, 180), [180, 190) and [190, 200) (Fig. 1a). The portion of chromosome Y reads, representing the fetal fraction, were calculated for each bin, respectively, which were then compared to that in the pooled data of the 10,000 NIPS results. In bins of [100, 110), [110, 120), [120, 130), [130, 140), [140, 150) and [150, 160), the mean fetal fractions were shown to increase by 1.98, 2.42, 2.64, 2.59, 2.01 and 1.44 times comparing to the average fetal fraction in pooled data. These results demonstrate in cfDNA fragments < 160 bp, the fetal fraction is significantly higher than that in the total cfDNA fragments in the maternal peripheral blood. As a result, it is possible to increase the cffDNA fraction by analyzing only sequences < 160 bp.

In order to achieve cffDNA enrichment, we developed our customized beads with three different concentrations (1.2×, 1.3× and 1.5× ratio) to samples. To evaluate the effect of our enrichment procedure, 174 blood samples from pregnancies with male fetus were tested using our enrichment procedure, and the portions of short reads (< 160 bp), long reads (≥ 160 bp) and the chromosome Y reads were calculated. Among them, 55 samples, 74 samples and 45 samples were processed using 1.2×, 1.3× and 1.5× ratio of beads, respectively. While the other 133 samples were processed without beads enrichment. Sequencing results revealed the distribution of read lengths altered (Fig. 1b). The portion of short DNA fragment (< 160 bp) was significantly increased using the beads with all three concentration (Fig. 1c). Importantly, the reads ratio of chromosome Y were also increased by 1.5–4 times, most significantly in beads with 1.5× ratio (Fig. 1d). These results proved that our enrichment procedure using 1.5× ratio of beads can effectively increase the portion of cffDNA comparing to that without enrichment

Since the procedure of cffDNA enrichment would discard most long cfDNA fragments, we quantified the amounts of sequencing libraries before and after enrichment, and found an approximately 91.76% reduction of input cfDNA would occur, which is a major concern of this method. For this reason, we tested whether the reduction of input DNA could impair the original library complexity, which represented the copies of whole genome DNA in cfDNA. Duplicated reads were considered as a good evaluation index to assess the robustness of the library. Lower rate of duplication represented higher complexity of input DNA, leading to a more reliable NIPS result. In order to find out the minimal threshold of input DNA complexity, we performed a computer simulation: first, we built a virtual input DNA pool with 0.1 to 100 copies of whole genome DNAs (library complexity ranged from 0.1 to 100). Then, we randomly sampled the reads with the size of 150 bp, which is close to the average cfDNA fragment size in maternal plasm, from virtual input DNA pools in different sequencing depth, ranged from 0.1× to 1×. Finally, we counted the duplication rate in different combinations of input DNA complexity and sequencing depth. The result revealed a low duplication rate with limited variation can be achieved with sequencing depth of 0.1× when input DNA complexity is higher than 3, which can be considered as the minimal input DNA complexity threshold for NIPS (Additional file 1: Figure S1). Furthermore, we developed a SNP-based method to compute the DNA complexity on 12 plasma samples (600 μL) before and after cffDNA enrichment. Although the DNA complexity reduced from 323.69 ± 77.42 to 84.09 ± 61.37 after enrichment, this number is still significantly higher than the threshold of required complexity of 3 in all the samples tested (Additional file 2: Table S1). In all, we proved that although cffDNA enrichment could reduce the input DNA complexity, our procedure can still obtain fully adequate DNA from maternal plasma for performing NIPS.

### Performance of NIPS using cffDNA enrichment on 1404 clinical samples

To evaluate the performance of NIPS with cffDNA enrichment, we recruited and restored plasma samples from 1404 women with singleton pregnancies who chose to have NIPS from Jan. 2017 to Feb. 2017 in our center. The clinical information is summarized in Additional file 3: Table S2. We retrospectively performed NIPS with cffDNA enrichment on these samples. We also collected the original ordinary NIPS results, as well as the confirmatory diagnostic results and the follow-up information, for further comparison.

**Fig. 2:**
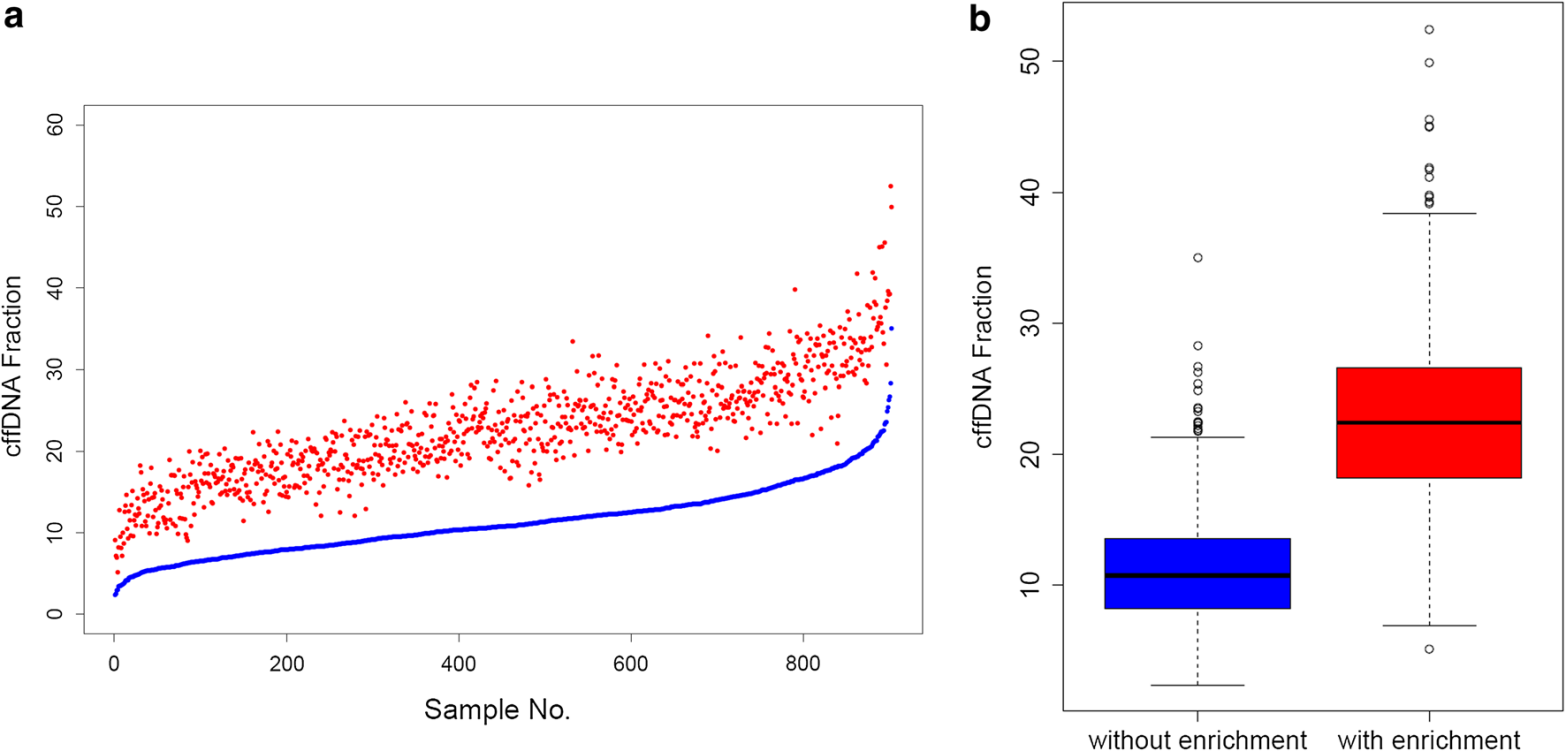
Comparison between the cffDNA fractions from new NIPS method and the ordinary method tested on the 902 pregnancies with male fetus. **a** cffDNA fractions of the 902 samples using the two NIPS methods. Blue dots represented the sorted cffDNA fractions from ordinary NIPS, while red dots represent that of the corresponding samples using NIPS method with enrichment. **b** Boxplot for cffDNA fraction between the two NIPS methods

We first selected the 902 samples with male fetuses from the 1404 samples and calculated their fetal fractions by reads ratio of chromosome Y. Original NIPS results revealed an average cffDNA fraction of 11.3 ± 4.2%, while the fetal fraction from the results of NIPS with cffDNA enrichment increased to 22.6 ± 6.6% (Fig. 2). To testify whether the elevated fetal fraction ensures a better performance on clinical samples, we compared the 1404 results of the new NIPS with cffDNA enrichment to the original NIPS results, as well as the confirmatory diagnostic results and the follow-up information. These results were summarized in Table 1. In brief, the original NIPS results showed a sensitivity of 100% (5/5), a specificity of 99.8% (1394/1397) and a positive predictive value (PPV) of 62.5% (5/8). While the new NIPS method showed a sensitivity of 100% (5/5), a specificity of 99.6% (1394/1399) and a PPV of 50% (5/10) (Table 1). Notably, the test-failure rate was significantly reduced in the new NIPS results (0.1% vs 0.7%, P < 0.01) (Additional file 4: Table S3).

**Table 1:** Comparison between results of NIPS with and without cffDNA enrichment on 1404 clinical samples

Method	NIPS without cffDNA enrichment	NIPS with cffDNA enrichment
Total	1404	1404
Test failure	10 (0.7%)	1 (0.1%)
Test positive	8	10
True positive	5	5
Test negative	1394	1394
True negative	1397	1399
Sensitivity	100% 95% CI (56.55–100)	100% 95% CI (56.55–100)
Specificity	99.79% 95% CI (99.37–99.93)	99.64% 95% CI (99.17–99.85)
PPV	62.50% 95% CI (30.6–86.3)	50% 95% CI (23.7–76.3)
NPV	100% 95% CI (99.73–100)	100% 95% CI (99.73–100)

*NIPS* noninvasive prenatal screening, *cffDNA* cell-free fetal DNA, *PPV* positive predictive value, *NPV* negative predictive value

### Performance of NIPS using cffDNA enrichment on 11 false negative samples

In clinic, the cause of false negative in NIPS is closely associated with low fetal fraction. To testify whether the new method could avoid the false negative results, a total of 11 restored false negative plasma samples from more than routine 100,000 ordinary NIPS cases were collected. By using NIPS with cffDNA enrichment, the fetal fractions were significantly increased by 1.9–2.7 times (P < 0.01). Importantly, 5 out of the 11 samples (45%) returned a positive result using the new method (Table 2), and these 5 positive results were consistent with the confirmatory karyotyping results of the fetuses. Our data demonstrated the cffDNA enrichment can effectively reduce the false negative rate of NIPS.

**Table 2:** Performance of NIPS using cffDNA enrichment on 11 false negative samples

No.	Karyotype	NIPS without cffDNA enrichment		NIPS with cffDNA enrichment	
		Fetal fraction (%)	Z score	Fetal fraction (%)	Z score
FN1	47,XX,+ 21	5.6	2.41	15.6	11.92^a^
FN2	47,XY,+ 18	8.5	− 0.41	18.6	− 1.05
FN3	47,XX,+ 21	4.8	1.14	11.4	5.076^a^
FN4	47,XY,+ 21	12.4	0.791	26.4	2.16
FN5	47,XY,+ 18	7.1	0.313	15.2	1.687
FN6	47,XY,+ 18	5.1	1.713	11.8	4.01^a^
FN7	47,XY,+ 21	11.2	0.163	18.8	− 1.86
FN8	47,XY,+ 21	9.2	− 0.56	19	1.096
FN9	47,XY,+ 21	7.5	− 0.16	14.7	1.634
FN10	47,XY,+ 21	6.4	2.031	14.6	10.14^a^
FN11	47,XY,+ 21	4.1	1.255	7.9	6.972^a^

*NIPS* noninvasive prenatal screening, *cffDNA* cell-free fetal DNA, *FN* false negative

^a^Positive results using cffDNA enrichment method

## Discussion

In this study, we developed a working experimental method for cffDNA enrichment, which can effectively increase the mean cffDNA fraction and obtain fully adequate cfDNA for NIPS. By retrospectively performing our new method on the 1415 clinical samples including 1404 routine clinical NIPS samples and 11 false negative NIPS samples, we were able to improve the test quality in the way of reducing test-failure rate and false negative rate.

To date, several attempts have been reported for fetal cfDNA enrichment in NIPS technology. Qiwei Yang et al. reported a PCR-based enrichment method to selectively amplify the fetal cfDNA [20]. Another work from Joaquim Vong et al. reported a single-strand DNA library preparation method to enrich the short cfDNA in maternal plasma [21]. Moreover, Stephanie Yu et al. reported a size-based method instead of count-based method for NIPS recently, which also showed a promising ability in detecting common trisomies [22]. These works all focused on the size difference of the cffDNA in maternal plasma to make progress in NIPS. However, evaluation on the feasibility of these methods still need validation by sufficient clinical samples. In this study, to ensure a reliable result, clinical samples with detailed follow-up information, including the fetal karyotype/CMA results or postnatal interview, were used to validate our new NIPS method.

The existence of discordant results, including false positive and false negative, has been regarded as one of the major limitations in NIPS [23]. In clinic, invasive confirmatory diagnosis will be recommended to all the NIPS positive cases to minimize the adverse effects of false positive results. While the false negative cases would often cause harmful consequences. Therefore, it is critical to uncover the underlying mechanisms of these false negatives results and improve the testing method accordingly. It is widely accepted that the false negative NIPS result is closely associated with low fetal fraction and true fetal mosaicism (TFM) [23]. Although most current NIPS protocol set up a fetal fraction threshold of 4% for the reliable testing result, there is still a chance of false negative when the fetal fraction is low, such as slightly higher than 4%. As suggested in the previous literature, all false negative NIPS cases are recommended to undergo fetal cfDNA enrichment, which can help to identify low fetal fraction as the potential cause [24]. Our results showed about half of the false negative cases (5/11) could be avoided by the new NIPS method. Interestingly, the 5 corrected false negative samples have relatively lower fetal fractions (4.1–6.4%) than the other 6 samples (7.1–12.4%), which also indicated the false negative results in these 5 cases could be due to the low fetal fraction. While the other 6 false negative cases may associate with other causes, such as TFM. However, the placental tissue, which is not available in these cases, is required for the confirmation of TFM.

By using our method with cffDNA enrichment, we were able to avoid 9 test-failure results, compared to ordinary NIPS method on the 1404 clinical samples. Importantly, our clinical follow-up information confirmed those cases with original test-failure results were all true negative. In clinical circumstance, cases with ‘no-call’ result were recommended a blood re-draw and another test, but no successful result can be guaranteed. Avoiding test-failures will result in a shorter turnaround time as well as a lower cost for the test, which should also generate less anxiety for the women.

One downside of our strategy is the elevated overall false-positive rates. In this study, there were 2 additional false positive cases among the results of the 1404 clinical samples. As confined placental mosaicism is considered as the major factor causing false positive [23], the placental chromosomal abnormalities could be amplified with cffDNA enrichment. However, our results indicated the decreased specificity (99.8% vs 99.6%) and increased false positive rate (0.6% vs 0.7%) in the test cohort is limited, and still comparable to other studies [25]. A larger scale validation of this method would be favorable in the future. Another disadvantage is that the original fetal fraction information would be lost using the new method. It is reported the level of fetal fraction could be related to some pregnancy complications, such as spontaneous preterm delivery, intrauterine growth retardation (IUGR) and pre-eclampsia in asymptomatic pregnant women, suggesting its potential diagnostic value [26–28]. While after fetal cfDNA enrichment, the original fetal fraction information is no longer available. Although this is beyond the scope of this study, further research may be addressed to determine whether there is any association between the original fetal fraction, the enriched fetal fraction and pregnancy complications.

It is important to accurately estimate the fetal fraction in NIPS. Previously, cffDNA levels were examined by qPCR [1]. When massive parallel sequencing is performed on both the maternal and cffDNA in a given plasma sample, the fetal fraction can be determined by examining genetic elements that differ between maternal and fetal DNA [29], including Y chromosomal markers [30], polymorphic markers [31], DNA methylation markers [32], and size-, count- and nucleosome profile-based methods [22, 33]. Genes on the Y chromosome are the most commonly used distinguishing marker [11, 12], but this method can be only used in pregnancies with male fetuses. Of note, cfDNA in maternal plasma is readily digested into small fragments by natural processes. Because of the small size of these fragments, no additional shearing is required before sequencing. Meanwhile, the fetal and maternal derived DNA fragments exhibit a difference in the distributions of size peaks [15]. Based on these characters, size-based estimation of fetal DNA fraction was established for the pregnancies with both male and female fetuses, that was also performed in this and our previous paper [34].

Although it is still controversial to expand the scope of NIPS at present, the technology to use cfDNA for detecting fetal copy number variations (CNVs) and single gene disorders has been developed for years. Several groups utilized whole genome sequencing, SNP-based and targeted sequencing of maternal plasma DNA and showed its huge potential for the detection of fetal microdeletion/microduplication syndromes [35–38]. However, the core statistical procedure is to compare the reads dosage on the target region between testing sample and normal controls, which is highly dependent on the cffDNA fraction. Previous studies showed that the minimal cffDNA fraction requirement for this purpose was 10% [34]. In addition, the cfDNA-based NIPS for single-gene disorders is much more challenging, because the cfDNA in maternal plasma is generally of minor population, hampering the reliable deduction of the maternal inherence of pathogenic variants at single-nucleotide resolution. Technologically, the development of relative haplotype dosage analysis (RHDO), which utilizes information regarding parental haplotypes flanking the variants of interest, has been demonstrated to greatly improve the accuracy of single-gene disorder detection [39]. Therefore, our method of cffDNA enrichment could contribute in expanding NIPS to the prenatal detection of fetal CNVs and single gene disorders in the future.

## Conclusions

This study demonstrates that it is feasible to increase the cffDNA fraction by selectively enriching short cfDNA fragments. Although the use of cffDNA enrichment in NIPS slightly decreased the specificity, this new method can avoid most test-failure results and reduce near half of false negative results caused by low fetal fraction, which improves the overall performance of NIPS in clinic. Our data also suggests the feasibility of using NIPS in detecting CNVs and single gene disorders in the future.

## Additional files

**Additional file 1: Figure S1.** Ratio of duplicated reads from computer simulation in the given conditions of library complexity and sequencing depth.

**Additional file 2: Table S1.** Comparison of library concentration and complexity before and after cffDNA enrichment.

**Additional file 3: Table S2.** Demographics of the 1404 clinical cases.

**Additional file 4: Table S3.** Information of ‘no-call’ results.

## Abbreviations

NIPS: noninvasive prenatal screening
cffDNA: based on cell-free fetal DNA
cfDNA: cell-free DNA
LOESS: locally weighted scatterplot smoothing
CMA: chromosomal microarray analysis
PPV: positive predictive value
TFM: true fetal mosaic
IUGR: intrauterine growth retardation
CNVs: copy number variations
RHDO: relative haplotype dosage analysis
