# Supplementary figures and images for: An enrichment method to increase cell-free fetal DNA fraction and significantly reduce false negatives and test failures for non-invasive prenatal screening: a feasibility study
Source: J Transl Med. 2019 Apr 11;17:124. doi: 10.1186/s12967-019-1871-x (PMC6460836; doi:10.1186/s12967-019-1871-x)

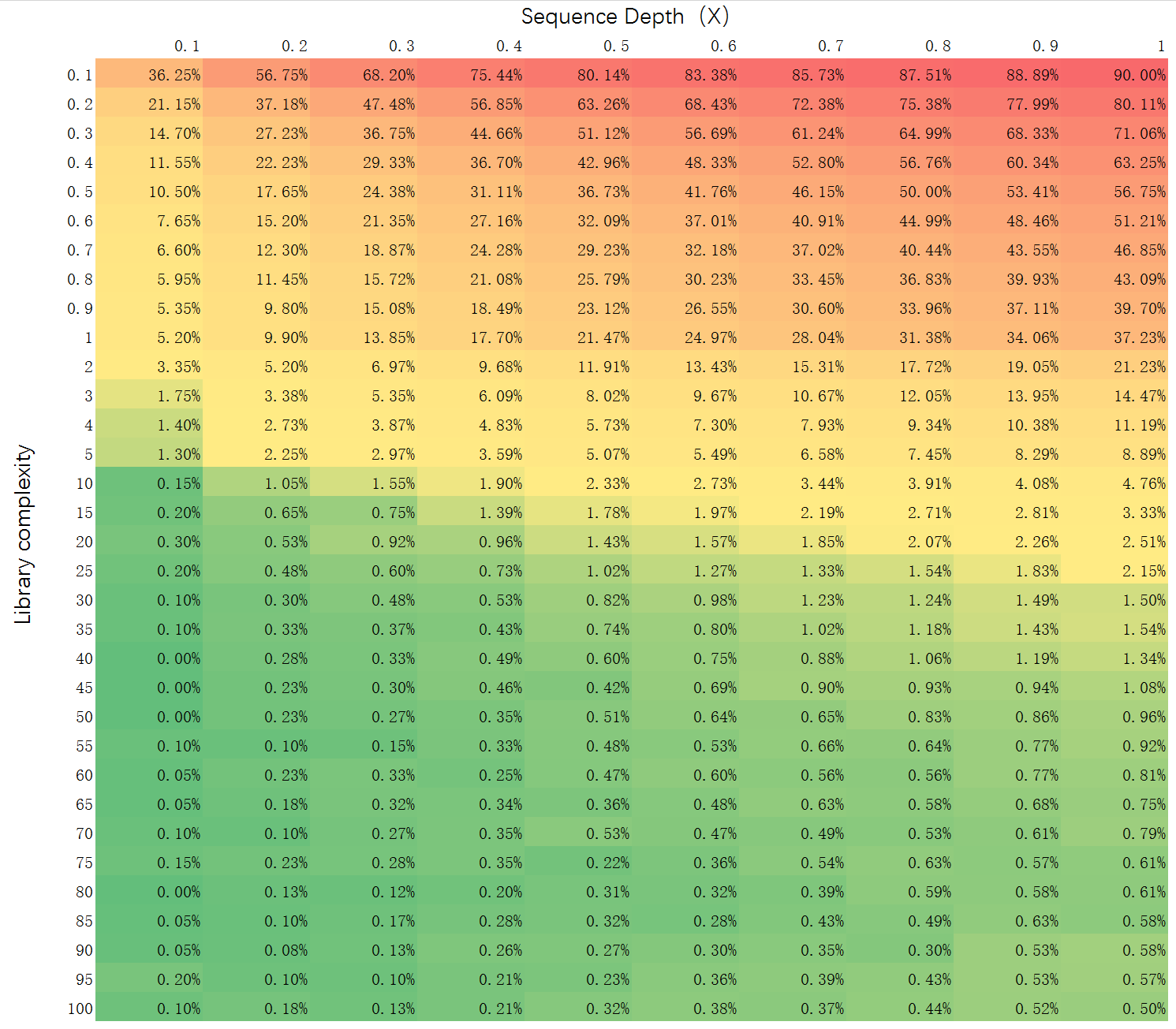

Supplement: Supplementary file 1 — Additional file 1: Figure S1. Ratio of duplicated reads from computer simulation in the given conditions of library complexity and sequencing depth. [file 12967_2019_1871_MOESM1_ESM.tif]
