# Supplementary material for: An enrichment method to increase cell-free fetal DNA fraction and significantly reduce false negatives and test failures for non-invasive prenatal screening: a feasibility study
Source: J Transl Med. 2019 Apr 11;17:124. doi: 10.1186/s12967-019-1871-x (PMC6460836; doi:10.1186/s12967-019-1871-x)
Supplement: Supplementary file 2 — Additional file 2: Table S1. Comparison of library concentration and complexity before and after cffDNA enrichment. [file 12967_2019_1871_MOESM2_ESM.docx]

Table S1 Comparison of library concentration and complexity before and after cffDNA enrichment.

|  | Before enrichment | | After enrichment | |
| --- | --- | --- | --- | --- |
| Sample ID | Library Concentration  (nmol/l) | Library Complexity  (×) | Library Concentration  (nmol/l) | Library Complexity  (×) |
| 5172 | 303.94 | 444.30 | 42.23 | 194.80 |
| 4849 | 309.78 | 465.40 | 11.29 | 22.56 |
| 5028 | 347.78 | 275.82 | 22.01 | 48.32 |
| 5100 | 369.03 | 310.01 | 45.07 | 175.59 |
| 5062 | 297.48 | 343.55 | 16.88 | 38.32 |
| 5141 | 464.13 | 265.74 | 13.82 | 30.86 |
| 5123 | 254.08 | 187.62 | 12.35 | 48.73 |
| 5173 | 272.78 | 297.89 | 20.31 | 58.30 |
| 5006 | 317.15 | 385.09 | 19.07 | 54.36 |
| 5099 | 322.84 | 296.49 | 41.57 | 157.94 |
| 5155 | 323.27 | 325.83 | 22.62 | 54.36 |
| 5126 | 246.93 | 286.59 | 39.39 | 124.94 |
