# Supplementary material for: An enrichment method to increase cell-free fetal DNA fraction and significantly reduce false negatives and test failures for non-invasive prenatal screening: a feasibility study
Source: J Transl Med. 2019 Apr 11;17:124. doi: 10.1186/s12967-019-1871-x (PMC6460836; doi:10.1186/s12967-019-1871-x)
Supplement: Supplementary file 3 — Additional file 3: Table S2. Demographics of the 1404 clinical cases. [file 12967_2019_1871_MOESM3_ESM.docx]

Table S2 Demographics of the 1404 clinical cases.

| Cohort size | 1404 |
| --- | --- |
| Maternal age, y |  |
| Mean | 31.6±4.8 |
| Median | 31.0 |
| Range | 18.0-44.0 |
| Gestational age, wk |  |
| Mean | 17.8±2.5 |
| Median | 17.6 |
| Range | 12.0-26.4 |
| Maternal body mass index (BMI), kg/m^2^ |  |
| Mean | 23.0±3.1 |
| Median | 22.7 |
| Range | 16.0-38.3 |
