# Supplementary material for: An enrichment method to increase cell-free fetal DNA fraction and significantly reduce false negatives and test failures for non-invasive prenatal screening: a feasibility study
Source: J Transl Med. 2019 Apr 11;17:124. doi: 10.1186/s12967-019-1871-x (PMC6460836; doi:10.1186/s12967-019-1871-x)
Supplement: Supplementary file 4 — Additional file 4: Table S3. Information of ‘no-call’ results. [file 12967_2019_1871_MOESM4_ESM.docx]

Table S3 Information of ‘no-call’ results.

| No. | Maternal  age | Gestational week | NIPS without  cffDNA enrichment | |  | NIPS with  cffDNA enrichment | |
| --- | --- | --- | --- | --- | --- | --- | --- |
|  |  |  | Fetal fraction  (%) | Result |  | Fetal fraction  (%) | Result |
| N1 | 27 | 19,1 | 7.52 | No-call |  | 19.73 | Negative |
| N2 | 27 | 19,4 | 5.18 | No-call |  | 17.42 | Negative |
| N3 | 35 | 19,2 | 4.22 | No-call |  | 13.79 | Negative |
| N4 | 27 | 19,2 | 1.5 | No-call |  | 13.64 | Negative |
| N5 | 39 | 16,0 | 6.94 | No-call |  | 13.82 | Negative |
| N6 | 35 | 16,1 | 7.01 | No-call |  | 15.49 | No-call |
| N7 | 31 | 17,1 | 3.98 | No-call |  | 9.8 | Negative |
| N8 | 40 | 16,1 | 5.72 | No-call |  | 15.47 | Negative |
| N9 | 38 | 17,0 | 2.38 | No-call |  | 9.09 | Negative |
| N10 | 35 | 14,0 | 6.14 | No-call |  | 14.38 | Negative |

NIPS, noninvasive prenatal screening; cffDNA, cell-free fetal DNA.
